# CITA GO-ON study. A community based multidomain lifestyle intervention to prevent cognitive decline. Protocol design and recruitment process

**DOI:** 10.3389/fnagi.2025.1539711

**Published:** 2025-06-16

**Authors:** Mikel Tainta, Mirian Ecay-Torres, Myriam Barandiaran, Ainara Estanga, Carolina López, Miren Altuna, Ane Iriondo, Jon Saldias, Maite Garcia-Sebastian, Marta Cañada, Maria de Arriba, Imanol Reparaz-Escudero, Mikel L. Sáez de Asteasu, Mikel Izquierdo, Nekane Balluerka, Arantxa Gorostiaga, Naia Ros, Goretti Soroa, Jara Domper, Lucia Gayoso, Maria Arrizabalaga-Lopez, Usune Etxeberria, Maria Ines Torres, Elena Alberdi, Estibaliz Capetillo-Zarate, Maider Mateo-Abad, Itziar Vergara, Javier Mar, Pablo Martinez-Lage

**Affiliations:** ^1^Centre for Research and Memory Clinic, CITA-alzheimer Foundation, Donostia-San Sebastián, País Vasco, Spain; ^2^Osakidetza, Organización Sanitaria Integrada (OSI) Goierri-Urola Garaia, Goierri-Urola Garaia, País Vasco, Spain; ^3^Instituto de Investigación Sanitaria Biogipuzkoa, Donostia-San Sebastián, País Vasco, Spain; ^4^Speech Interactive Research Group, University of the Basque Country UPV/EHU, Leioa, País Vasco, Spain; ^5^Osakidetza, Organización Sanitaria Integrada (OSI) Donostialdea, Donostialdea, País Vasco, Spain; ^6^Osakidetza, Organización Sanitaria Integrada (OSI) Debabarrena, Debabarrena, País Vasco, Spain; ^7^Navarrabiomed, Hospital Universitario de Navarra (HUN)-Universidad Pública de Navarra (UPNA), IdiSNA, Pamplona, Navarra, Spain; ^8^Centro de Investigacion Biomedica en Red of Frailty and Healthy Aging (CIBERFES), Instituto de Salud Carlos III, Madrid, Spain; ^9^GOe Tech Center, Technology Center in Gastronomy, Basque Culinary Center, Donostia-San Sebastián, País Vasco, Spain; ^10^Basque Culinary Center, Faculty of Gastronomic Sciences, Mondragon Unibertsitatea, Donostia-San Sebastián, País Vasco, Spain; ^11^Achucarro Basque Center for Neuroscience, Leioa, País Vasco, Spain; ^12^Department of Neurosciences, Faculty of Medicine and Nursery, University of the Basque Country UPV/EHU, Leioa, País Vasco, Spain; ^13^Department of Neurosciences, Faculty of Pharmacy, University of the Basque Country UPV/EHU, Vitoria-Gasteiz, Spain; ^14^IKERBASQUE, Basque Foundation for Science, Bilbao, País Vasco, Spain

**Keywords:** cognitive impairment, dementia prevention, lifestyle, multidomain intervention, randomized trial data collection

## Abstract

**Introduction:**

Growing research suggests that dementia is a complex disorder with multiple risk factors and causes. The Finnish Geriatric Intervention Study to Prevent Cognitive Impairment and Disability (FINGER) demonstrated that lifestyle interventions could confer cognitive benefits. Inspired by this, the GOIZ-ZAINDU (GZ) feasibility study adapted the FINGER approach to the Basque context. Building upon the GZ study, the CITA GO-ON trial aims to enhance and expand the evidence supporting dementia prevention through a multidomain intervention of risk factor management and resilience promotion.

**Methods:**

It is a two-year, population-based, randomized controlled trial to prevent cognitive decline in adults aged 60–85 years with Cardiovascular Risk Factors, Aging and Dementia (CAIDE) risk score ≥6, no dementia, and below-than-expected performance on at least one of three cognitive screening tests. Participants are randomized (1:1) to receive either Regular Health Advice (RHA) or a Multidomain Intervention (MD-Int) that encompasses cognitive training, socio-emotional skills, multicomponent physical exercise, nutritional and culinary intervention, and monitoring for cardiovascular risks, pharmacological drug mismanagement, and comorbidities. The primary outcome is the efficacy of the intervention to reduce the risk of cognitive decline measured by the global composite *z*-score of the modified Neuropsychological Test Battery over two years. The secondary outcomes measure cost-effectiveness, quality of life, and functional abilities. Blood samples and brain imaging will also be collected to evaluate the effects of the intervention on brain structure and plasma biomarkers.

**Results:**

Recruitment has been completed with 1051 participants selected (mean age (standard deviation, SD) of 69.65 (6.36), 58,50 % female, and mean CAIDE (SD) of 7.62 (1.427). The final participant is expected to complete the last study visit by the autumn of 2026.

**Discussion:**

The CITA GO-ON Study, as a part of the World-Wide FINGERS network, is designed to validate the efficacy of a multidomain lifestyle intervention for dementia prevention and contribute valuable data to inform public health strategies fostering healthy, active aging.

## 1 Background

Dementia and late-life cognitive impairment impose significant disability and degradation of the quality of life for both affected individuals and their caregivers, leading to elevated social and healthcare expenditures. The global number of persons with dementia has doubled in recent years (GBD 2016 Dementia Collaborators, 2019), and forecasting models project a marked escalation in dementia cases if effective preventive measures are not established (Soto-Gordoa et al., 2015).

Investigations into dementia and neurodegenerative conditions have uncovered a multitude of underlying coexisting comorbidities, particularly in older populations (Larson et al., 2013; Wu et al., 2017). In addition, a spectrum of lifestyle factors has been identified as influential in the risk trajectory for cognitive decline throughout life (Norton et al., 2014). Observational studies have reinforced that addressing traditional cardiovascular risk elements, such as hypertension, obesity, and diabetes, may delay the onset of cognitive deficits (Kemppainen et al., 2018; O’Brien et al., 2019; Dhana et al., 2020). A cognitively and physically active lifestyle is associated with healthy cerebral aging. Recent insights also revealed that deficits in social engagement and emotional well-being may contribute to the emergence of cognitive impairment (Fratiglioni et al., 2020; Lisko et al., 2021; Röhr et al., 2023). The multifactorial nature of cognitive decline suggests that interventions targeting a combination of lifestyle and health factors might offer superior preventive capability (Solomon et al., 2013; Kivipelto, 2016). While some pharmacological trials have shed light on the treatment of Alzheimer’s dementia (Mintun et al., 2021; van Dyck et al., 2022), a significant number have not successfully demonstrated efficacy in delaying cognitive decline (Kivipelto et al., 2018a), highlighting the need for complementary nonpharmacological strategies for both primary and secondary prevention. The Lancet Commission on Dementia Prevention, Intervention, and Care underscores the importance of initiatives that promote lifestyle alterations and mitigate dementia risk factors in midlife and beyond (Livingston et al., 2020).

The Cardiovascular Risk Factors, Aging, and Dementia (CAIDE) risk score (Kivipelto et al., 2006) is a well-known and validated tool that can predict cognitive trajectories. The Finnish Geriatric Intervention Study to Prevent Cognitive Impairment and Disability (FINGER) trial (Ngandu et al., 2015) is a large-scale investigation that substantiates the cognitive benefits of a multidomain lifestyle intervention for older adults at elevated risk of dementia, as determined by the CAIDE dementia risk score (Ngandu et al., 2014). Other similar multidomain interventions, such as the Multidomain Alzheimer Preventive Trial (MAPT) (Andrieu et al., 2017) and Prevention of Dementia by Intensive Vascular Care (van Charante et al., 2016) (preDIVA) studies, yielded positive outcomes within participant subgroups characterized by higher CAIDE risk scores or those with cerebral amyloid deposition. The World-Wide FINGERS network (Kivipelto et al., 2020) was established to advance scientific substantiation of this complex domain. This initiative is dedicated to enhancing protocol standardization and facilitating data exchange to bolster research efficacy.

**Figure 1:**
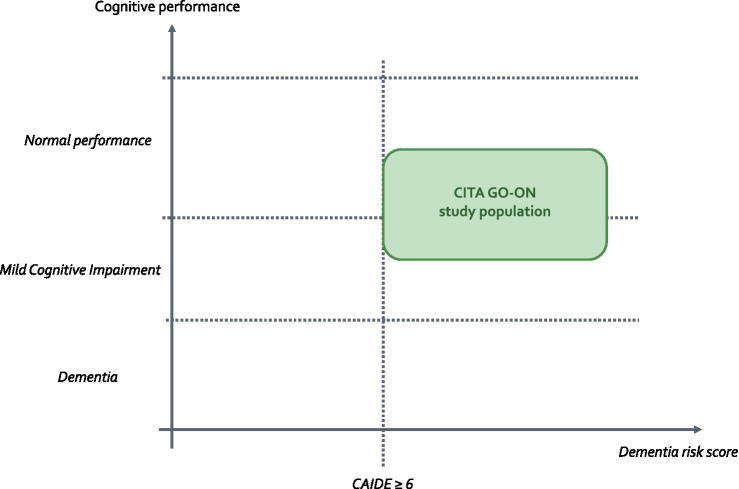
CITA GO-ON study target population.

In the spirit of the pioneering FINGER study, the CITA-alzheimer research team group applied the CAIDE dementia risk score in non-demented longitudinal cohorts and population-based investigations (Ecay-Torres et al., 2018). Our findings revealed that diminished cognitive performance was correlated with CAIDE risk score, vascular, amyloid, and neurodegenerative biomarkers in asymptomatic at-risk adults. Recently, our team embarked on a feasibility study called the GOIZ ZAINDU (GZ) trial (Tainta et al., 2024a), which adapted the FINGER multidomain lifestyle intervention model to the cultural, healthcare, and social frameworks of the Basque region. Adherence to the intervention program and integration with existing health system protocols were pivotal in attaining the primary objective. Efficacy analysis revealed a significant less decline in executive function and processing speed in the multidomain intervention (MD-Int) group. These pilot findings laid the groundwork for a comprehensive efficacy trial, the “CITA GO-ON” study, registered with ClinicalTrials.gov (NCT04840030). This trial encompasses older adults approaching cognitive decline, characterized by an augmented dementia risk as assessed by the CAIDE score manifesting either suboptimal performance in brief cognitive evaluations or persistent memory complaints (Figure 1).

The primary objective of the CITA GO-ON study is to evaluate the efficacy of a multidomain lifestyle intervention as a preventive strategy against cognitive decline. Concurrently, this study will establish a repository of brain imaging and biomarker data, facilitating exploratory research on the physiological impacts of lifestyle modifications in the adult population. This paper delineates the principal protocol of the CITA GO-ON study, outlines the methodology used for participant recruitment, and provides an update on the current status of the study plan.

The success of the FINGER trial may be attributed to the meticulous selection of participants.

This encompasses an evaluation of the intervention cost-effectiveness and its potential integration into public health frameworks aimed to promote healthy and active aging, as well as dementia prevention.

This hypothesis is based on the premise that such interventions attenuate modifiable risk factors and generate positive neuroplastic changes, as demonstrated by brain imaging and biomarker profiles. These results are expected to be attained at an acceptable cost-effectiveness to ensure the public health utility of the intervention in promoting healthy aging and dementia prevention.

The central hypothesis of the CITA GO-ON study postulates that a multidomain lifestyle intervention over 2 years in older adults at high risk for dementia is significantly efficacious to reduce the risk of progression to cognitive decline.

## 2 Materials and methods

### 2.1 Study design

The CITA GO-ON study is a single-center, population-based, randomized controlled two year trial to evaluate the efficacy of a multidomain lifestyle and vascular risk factor intervention in averting cognitive decline.

### 2.2 Study objectives

The primary objective is to ascertain the efficacy of the intervention to attain a 20% reduction in the proportion of subjects exhibiting a decline in cognitive performance, as measured by the change in the global cognition z-score on the modified Neuropsychological Test Battery (mNTB) (Harrison et al., 2007; Kivipelto et al., 2013), between baseline and 24 month visits in the multidomain intervention group (MD-Int) relative to the regular health advice (RHA) group. Secondary objectives include: (1) analyzing the cost-effectiveness of the intervention; (2) determining the beneficial effects of the intervention on functional abilities, quality of life, and symptoms of depression and anxiety; and (3) investigating the impact of lifestyle interventions on: neuroimaging parameters of brain volumetry, white matter integrity and vascular burden; plasma biomarkers of Alzheimer’s pathology, neurodegeneration, and neuroinflammation; epigenetic marks; lipid dysregulation; and cognitive and brain reserve capacity.

### 2.3 Study participants

Eligible participants are non-demented older adults aged 60–85 years who are willing and able to engage with all study evaluations and interventions, have a CAIDE dementia risk score of 6 or higher, and score below the established cutoff points in at least one of three brief cognitive evaluations: Fototest score ≤ 35, Memory Alteration Test (T@M) score ≤ 40, (Carnero-Pardo and Montoro-Ríos, 2004; Rami et al., 2007; Tainta et al., 2024b), or score ≥20 on the first 12 items (episodic memory) of the Cognitive Change Index (CCI) self-report version (Saykin et al., 2006; Risacher et al., 2017). The exclusion criteria, detailed in Supplementary Table S1, comprise conditions that may hinder the attainment of study goals, adherence to procedures, as well as specific clinical conditions that could by themselves compromise cognitive performance.

### 2.4 Ethical approval and consent to participate

This study was approved by the Euskadi Drug Research Ethics Committee (CEIm-E) (ID: PI2020244). This randomized trial is been conducted following the Consolidated Standards of Reporting Trials guidelines (CONSORT) (Schulz et al., 2010). All participants provide written informed consent. Samples obtained and unanalyzed will be stored as biological samples after the corresponding informed consent is signed. Explicit consent for the publication of the study results and experience was obtained from each participant.

### 2.5 Screening period

The recruitment approach was community focused in Gipuzkoa, Spain. The primary recruitment strategy includes collaboration with local town halls and community services supported by press releases, social media campaigns, direct mailings, and community talks. Broad outreach efforts employ mass mailing to the target age demographics, local advertisements at bus stops, billboards, and local publications. This initial marketing phase was augmented with informative sessions conducted at neighborhood associations. Comprehensive study information and access to informed consent documents are available on the study website^1^.

#### 2.5.1 Prescreening

Persons interested fill out an online form that allows the identification of those who meet the pre-established requirements before attending an individualized face-to-face screening visit.

For the online pre-screening procedure interested participants access a web-based formulary [Research Electronic Data Capture (REDCap)-based; Harris et al., 2008, 2018] to collect basic sociodemographic data, a standardized questionnaire that allows to estimate a CAIDE dementia risk score and the presence of exclusion criteria (Table 1). Prior to the fulfillment of the on-line form (Supplementary Table S2), participants receive detailed information on the study objectives, future activities, and evaluations.

**Table 1:** Variables at prescreening and screening visits.

Screening variables		Prescreening (virtual evaluation)	Screening (face-to-face evaluation)
	Time for randomization visit	- 36 weeks	- 24 weeks
Personal data	Contact information	x	
	Sociodemographic data	x	
	Habit of using the internet and virtual communication tools.	x	
Dementia risk score	CAIDE	x	x
	Inclusion/Exclusion criteria	x	x
	Informed consent		x
Screening tools	Cognitive Change Index	x	
	Barthel index		x
	Geriatric Depression Scale		x
	Fototest, T@M		x
Physical condition	Anthropometry and cardiovascular risk evaluation		x
Medical history	Self-reported impairments and symptoms		x
	Reported medical conditions and current medication: information from participants’ GGPregarding current diagnoses, medication and lab values, vascular risk factors, and smoking habit.		x

#### 2.5.2 Screening visit

Participants with an estimated CAIDE dementia risk score ≥ six and no reported exclusion criteria are invited for an in-person selection evaluation visit and full written information is provided. At the beginning of the interview, information regarding the study details is provided to all participants. After reading the informed consent form and solving all possible questions and doubts, participants sign an informed consent. Subsequently, past medical history is recorded to rule out exclusion criteria. Demographic and anthropometry data together with cholesterol determinations are collected and a current CAIDE dementia risk score is obtained. Brief cognitive testing is then administered and CCI scores are accounted for to complete inclusion/exclusion criteria accomplishment. The variables obtained at the screening visit are depicted in Table 1.

### 2.6 Baseline evaluation

**Figure 2:**
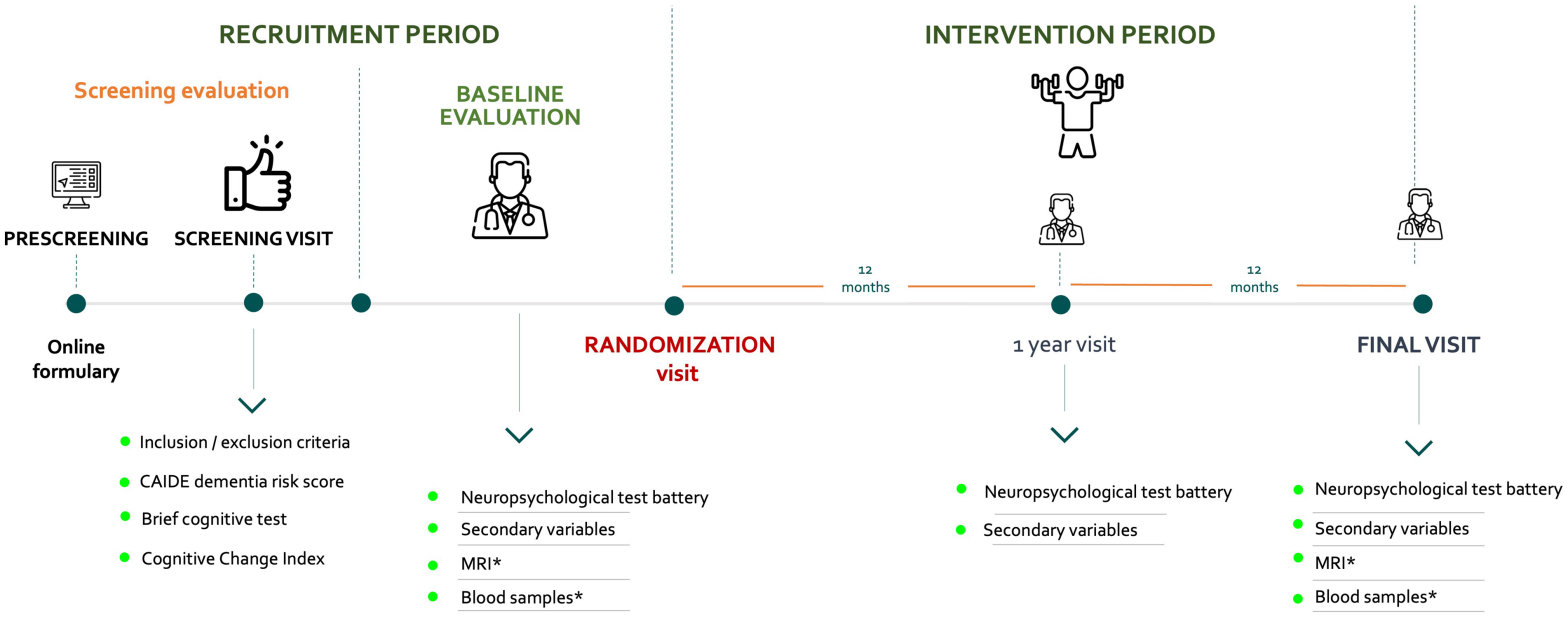
Timeline of the CITA GO-ON study periods. *Blood sample collection and neuroimaging acquisition are conducted in a subgroup of participants who have consented to it.

The recruitment period is defined as the time between the prescreening and the randomization visit (Figure 2). The CITA GO-ON study assessments and the corresponding schedule and procedures are detailed in Tables 2, 3. All participants complete the Baseline, 12-month, and 24-month visits for primary and secondary efficacy variables. In participants who consent, biological samples and neuroimaging (3T MRI) data are collected at baseline and 24-month follow-up visits.

**Table 2:** CITA GO-ON study assessments.

Primary outcomes	
Cognitive performance I	Modified Neuropsychological Test Battery (global composite z scores)
Secondary outcomes	
Cognitive performance II	mNTB specific cognitive domains: episodic memory, executive function, and processing speed.
Functional independence	Amsterdam Questionnaire for Instrumental Activities of Daily Living
Quality of Life	EuroQoL-5D
Psychological and behavioral eval. I	Goldberg Anxiety and Depression Scale (GADS)
Exploratory variables	
Cognitive performance III	Leisure and productive activities questionnaire. Visual Perception and Construction composite.
Socioemotional evaluation	Physiological stress (Heart rate variability, EMWAVE); Emotional attention; Emotional understanding; Emotional regulation (TMMS-22 Old); Mindful Attention Awareness Scale (MAAS; Barajas and Garra, 2014); Subjective perception of loneliness (Velarde-Mayol et al., 2016) (UCLA); Satisfaction with Life Scale (Pons et al., 2000) (SWLS).
Nutritional evaluation I	Mediterranean Diet Adherence Screener (Schröder et al., 2011) (MEDAS) questionnaire
Nutritional evaluation II	Food Frequency Questionnaire(de la Fuente-Arrillaga et al., 2010) (FFQ); Home Cooking Frequency Questionnaire (Goni et al., 2022) (HCFQ),
Physical activity and exercise eval. I	SPPB (Short Physical Performance Battery), Six meters walking speed
Physical activity and exercise eval. II	Dual-task 6 meters walking speed, 30” Chair stand test, Handgrip test and step up and go tests (recorded at basal visit, 12-month visit and the end of the study). Daily physical activity is measured by steps or meters per day.
PHYSICAL and METABOLIC CONDITION	Anthropometric measure (height, weight, calculation body-mass-index and waist and hip perimeter), blood pressure, glucose levels, total cholesterol.
Brain MRI	Structural and DTI imaging
Blood samples	Blood sample for plasma biomarkers and DNA extraction.
Cognitive performance IV	Cognitive Reserve Questionnaire (Rami et al., 2011); Bilingualism questionnaire (https://sites.la.utexas.edu/bilingual/)
Safety variables	
Adverse events evaluations	
Information from participants GP regarding current diagnoses and medication and lab values (blood count, cholesterol, glucose, renal and liver function, thyroid hormones, B12 vitamin, folic acid)	
Other control variables	
Psychological and behavioral eval. II	Perceived Stress (Levenstein et al., 1993) (PSQ), and sleep quality (Pittsburgh Quality of sleep scale).
Use of e-health systems to track health habits	

**Table 3:** CITA GO-ON study visits assessments.

Variables	Baseline evaluation	Randomization	v1	v2	v3	v4	v5	v6
Time from randomization visit	-12 weeks	0	4 months	8 months	12 months	16 months	20 months	24 months
Visit time window	+/− 30 d.		+/− 20 d.	+/− 20 d.	+/− 20 d.	+/− 20 d.	+/− 20 d.	window of 90 d.
Primary outcomes								
Cognitive performance I	x				x			x
Secondary outcomes								
Cognitive performance II	x				x			x
Functional independence	x				x			x
QoL	x				x			x
Psychological and behavioral eval. I and II	x				x			x
Exploratory variables								
Cognitive performance III	x				x			x
Socioemotional evaluation	x				x			x
Nutritional evaluation I	x				x			x
Nutritional evaluation II	x							x
Physical activity and exercise eval. I	x				x			x
Physical activity and exercise eval. II	x				x			x
PHYSICAL and METABOLIC CONDITION	x				x			x
Brain MRI	x							x
Blood samples	x							x
Cognitive performance IV	x							
Safety variables								
Adverse events evaluations	x				x			x
Information from participants GP regarding current diagnoses and medication and lab values.	x							x
Intervention group-specific variables								
Nutritional evaluation I			x	x		x	x	
Physical activity and exercise eval. I			x	x		x	x	
PHYSICAL and METABOLIC CONDITION			x	x		x	x	

#### 2.6.1 Primary outcome: cognitive performance I

In accordance with the WWFINGERS network data harmonization recommendations, cognitive performance change is the primary outcome. It is assessed using the modified Neuropsychological Test Battery (mNTB) (Harrison et al., 2007), previously described for in our pilot study (Tainta et al., 2024a) and in Supplementary Table S3. It is recorded by trained neuropsychologists who are blinded to the group assignment at baseline evaluation and at the 12-month and 24-month visits. The mNTB Cognitive Global score is obtained. Composite cognitive z-scores are calculated based on the results of all single tests. The higher the score, the better the performance. Changes in global cognition are assessed by differences between Global Cognitive composite z-scores at baseline and 24-month visits.

#### 2.6.2 Secondary outcome assessments

- *Cognitive Domains – (cognitive performance II):* mNTB executive function, Memory, and Processing speed scores are obtained. Apart from the mNTB scores, perception and construction abilities function composites are also assessed using the Judgement Line Orientation, Rey Complex Figure copy, and 15 object test. Differences in pre-and post-intervention scores in mNTB-episodic memory, mNTB-executive function, and mNTB-processing speed z-scores together with perception and construction abilities composites are used to assess changes in specific cognitive domains.
- *Functionality:* The Amsterdam Questionnaire for Instrumental Activities of Daily Living (A-IADL-Q) (Jutten et al., 2017) serves to assess the impact of the intervention on an individual’s functional capacity.
- *Quality of life:* The EuroQoL-5D (Kind et al., 1998) formulary evaluates participants’ quality of life.
- *Affective and behavioral evaluation:* The Goldberg Anxiety and Depression Scale (Goldberg et al., 1988) has been incorporated into the study design to evaluate the influence of the intervention on the symptoms of depression and anxiety.

#### 2.6.3 Exploratory assessment

A comprehensive assessment of the socioemotional domain, dietary and culinary habits, physical fitness, and vascular risk factors have been devised to evaluate the impact of the multidomain intervention (Table 2).

- **Neuroimaging collection:** Subjects who consent will undergo a brain MRI during Baseline and 24-month visit evaluation to evaluate brain volume trajectories, cortical thickness, and white matter changes (MRI). MRI sequences are listed in Table S4.
- **Biological sample collection and banking:** Biological samples are collected, processed, and stored at Baseline and at 24-month visits to evaluate the effect of the intervention on plasma biomarkers. Whole blood, buffy coats, plasma, and serum are collected in polypropylene tubes, transferred onto polypropylene cryovials, and immediately stored at-80°C for future analysis after centrifugation. The analyses for plasma AT (N) biomarkers will be performed on a SiMoA (Single Molecule Array) platform using commercial kits from Quanteryx APOE genotyping: APOE gene will be genotyped by Restriction Fragment Length Polymorphism (RFLP) technique (DNA amplification, specific primers, PCR, and enzymatic restriction with CfoI enzyme). Stool samples for microbiome analysis will also be collected.

#### 2.6.4 Safety assessments

Participants provide information on past medical history and current illnesses, which is confirmed through their clinical history obtained from their General Practitioners (GPs) at the beginning of the study period and follow-up visits. Any change in a participant health along the study is recorded as an adverse event. Adverse events are actively identified through structured interviews at every follow-up visit. Current diagnoses, medication, and lab values, including blood count, cholesterol, glucose, renal and liver function, thyroid hormones, B12 vitamin, and folic acid, are documented before randomization.

### 2.7 Intervention period

#### 2.7.1 Randomization visit

This in-person visit is conducted by the study neurologists, who review medical history, risk factors, cognitive symptoms, functional status, cognitive performance and perform a general and neurological physical exam. Individuals diagnosed with dementia, as defined by the DSM-IV, are excluded. A diagnosis of Mild Cognitive Impairment (MCI) (Petersen, 2004) is confirmed when applicable. Participants are then randomly assigned to the Group 1 or Group 2 to complete the randomization visit. Participants are then informed of their designated group and receive both verbal and written information and recommendations on active and healthy aging, control of risk factors dietary advice, physical activity, social engagement, and routine suggestions aimed at fostering cognitively and affectively stimulating practices.

#### 2.7.2 Group 1—RHA group

Participants assigned to the RHA group undergo follow-up evaluations at 12-month and 24-month marks. Adherence to the lifestyle recommendations provided during the randomization visit is self-directed, with no additional guidance on the activities pursued to follow these recommendations. Participants may connect with other healthcare professionals, including their general practitioners, who are not part of the study team according to their individual health needs.

#### 2.7.3 Group 2—MD-Int group

The MD-Int program represents an update of the GZ pilot study intervention program, introducing a socio-emotional component along with a greater frequency of visits and extended materials within each intervention domain. The program aims to promote healthy brain aging, provide tools for incorporating these routines into daily life, and strengthen the social environment. The program is tailored to each participant needs and abilities throughout the study. Apart from the 12-month and 24-month visits participants in the MD-Int group receive individual interviews with the study physician and physiotherapist every 4 months. In these visits cardiovascular and metabolic parameters (blood pressure, pulse, weight, BMI, hip and waist perimeters, capillary glucose, and cholesterol), Mediterranean diet adherence, physical capacity and activity, active medications and adverse events are reviewed and monitored.

The MD-Int program is administered in small groups of 10–15 participants, with a designated investigator or “group leader” overseeing the various MD-Int activities and intervention staff. Intervention components are listed in Table 4. Cognitive stimulation intervention comprises 16 group sessions in workshops (24 h) conducted by a neuropsychologist and over 100 h of individual at-home cognitive training exercises using the EXERCITA® program that was specifically designed after the pilot study. Nutritional and culinary intervention includes eight nutritional and show cooking workshops facilitated by study nutritionists and professional chefs. Individual recommendations for physical exercise based on the VIVIFRAIL©^2^ multicomponent physical exercise tool. And finally, up to 16 group workshops focused on promoting socio-emotional skills the intervention.

**Table 4:** Description of CITA GO-ON MD-Int components.

Intervention component	Description
Cognitive intervention	The targeted cognitive domains include attention, memory, language, executive function, perception, and visual construction abilities. 16 group workshops are distributed over six periods of 3 months throughout the study period. In addition, each participant will receive individually prescribed paper materials tailored to their cognitive status, educational level, and occupation level for cognitive training. These materials are designed to improve each cognitive domain and require a commitment of approximately 20 min of individual sessions three times per week. This initiative promotes cognitive functioning by incorporating cognitively stimulating habits and routines into daily life. Supplementary Figures S1–S3, show examples of routines discussed in the workshops to incorporate them into daily routines.
Physical exercise intervention	The objective is to improve muscle strength/power, cardiovascular endurance (i.e., walking), static and dynamic balance, and flexibility. The program is based on the WHO-recommended material of the VIVIFRAIL© toolkit (http://vivifrail.com/resources/), a home-based multicomponent tailored exercise program that can be implemented during unsupervised sessions with four progressive levels: Disability [A], Frailty [B], Pre-frailty [C], and Robust [D] or advanced fitness level [E-FIT]. The VIVIFRAIL Multicomponent Physical Exercise Program to Prevent Frailty and the Risk of Falls represents an excellent example of an evidence-based program. The VIVIFRAIL physical exercise guide (http://vivifrail.com/resources/) consists of lower-limb, upper-body, balance, and gait re-training exercises.
Nutritional and culinary intervention	The program aims to improve adherence to the Mediterranean Diet through educational workshops and individual counseling sessions. Eight workshops will be held over 2 years, each lasting 1.5–2 h, and are open to cohabitants of participants who do not cook at home. The workshops are conducted by nutritionists and chefs and provide health-related information and culinary education. The program also offers a guide outlining healthy recipes and nutritional information, as well as online and printed resources such as recorded workshops and video recipes to support participants in making healthy lifestyle changes.
Socioemotional intervention	This branch focuses on emotional well-being by fostering awareness, communication, and regulation of emotions. This is achieved by emphasizing emotional intelligence and the cognitive impact of emotions. This program encompasses six key modules: psychoeducation, emotional awareness, emotional communication, emotional management, coping with loneliness, and promotion of subjective well-being. The intervention will be implemented in 16 group sessions scheduled, along with cognitive intervention workshops. The group work is complemented by a homework assignment designed to integrate the session’s concepts into participants’ daily lives. Socioemotional intervention plays a transversal role in multicomponent interventions, enhancing social commitment, motivation, and adherence to the program.
Cardiovascular risk factor and comorbidities intervention	Every 4 months, the study team will evaluate the participants for cardiovascular risk factor check-ups, including measurements of blood pressure, weight, capillary blood glucose, and cholesterol levels. If the evaluation suggests the emergence of a new risk factor or poor control of a known risk factor, the primary care physician will provide appropriate advice and recommendations for the management, initiation, or adjustment of pharmacological treatments. Additionally, at each visit, the patient’s active medications will be reviewed following the current guidelines for minimizing inappropriate medications from the Basque Public Health System (http://www.osakidetza.euskadi.net/cevime, Vol 20 n° 8, 2012).

### 2.8 Adherence and participant retention

Adherence is a vital aspect in studies on lifestyle interventions that entail a significant degree of intricacy and impact on the social and familial structure functioning of participants. CITA GO-ON implements specific strategies to promote adherence. During the randomization phase, the study coordinator conducts a face-to-face or telephone/virtual interview with each MD-Int participant to explain all study activities, identify potential obstacles for compliance with programmed interventions and assessments, and schedule workshops and activities. Each participant is provided with a paper form or virtual record of compliance with the intervention activities and study contact information. Separate forms to account for adherence to cognitive intervention (attendance to workshops and completion of individual home exercises), socio-emotional (attendance to workshops and completion of individual home exercises), physical activities (self-reported completion of prescribed activities), risk factor control (attendance to follow-up visits), and dietary intervention (attendance to workshops) will be collected. This will allow separate analysis of the association of adherence to each intervention and efficacy. The group leader or study coordinator assesses this register at every follow-up visit. After each group session, time is dedicated to addressing any doubts, issues, or challenges the participants may have. Furthermore, group leaders review the group activity plan and schedule for subsequent study activities. The variables included in “Nutritional Evaluation I” and “Physical Activity & Exercise Evaluation I” are surrogate markers for self-reported adherence. In this sense, the Mediterranean Diet Adherence Screener, the MEDAS questionnaire, the SPPB, the 6 m walking speed test, cardiovascular factors (blood pressure), and body anthropometry, measured every 4 months, will help us understand if intervention activities are changing participants’ lifestyles and will help us detect nonadherence situations.

Participants are permitted to bring a close relative to the nutritional and show cooking group meetings, mainly if they are not responsible for preparing the daily meal menu at home cooking.

### 2.9 Virtual tools

All participants are questioned about previous experience with digital tools, including video calls, electronic mail and messages, and social media. During workshops, participants are trained to participate in group video calls and using these virtual tools to prepare for the possibility of utilizing them. The goal is to provide the necessary tools to evaluate and interact with participants who may be unable to attend in-person study visits. In fact, a telematic mNTB version has been administered in a sample of 50 persons before the CITA GO-ON study kick-off and will be administered via video call if necessary.

Self-administered REDCap questionnaires, mail reminders, and phone calls are used to communicate with the participants. Intervention materials, such as recommendations, recorded workshops, and individual cognitive training materials, are available on a web storage service platform for the intervention group.

### 2.10 Statistical considerations

#### 2.10.1 Sample size

Sample size calculations were based on the FINGER trial (Ngandu et al., 2015) and GZ pilot study data (Tainta et al., 2024a). Accepting a significance level of 5% and a statistical power of 90% in a one-sided test, 584 subjects would be necessary for each group, with a total of 1,096 participants, to detect a statistically significant difference between the two proportions of participants who decline in performance on the mNTB after 2 years, 49% for the RHA group and 32% for the MI group. The sample size has been calculated to allow a predicted 20% dropout rate according to the GZ study data. In the same way, considering the same level of significance, statistical power, and dropout rate, in order to detect a decline in performance on the mNTB score after 2 years, considering data from the GOIZ ZAINDU Pilot study, we calculated that a sample size of 802 randomized participants would be sufficient to detect by a two tailed Student t test, a difference between groups of 0.10 units in global mNTB score change, considering a 0.33 standard deviation.

#### 2.10.2 Allocation

Randomization list is created to follow a 1:1 ratio stratified by age, sex, and presence of mild cognitive impairment (MCI). To minimize bias, the allocation of participants to each group will be concealed through the REDCap randomization procedure, previous to the recruitment process begins. A member of the research team who will not carry out primary outcome assessments will enter participant details into the randomization system and inform relevant parties of the outcome of group allocation. The study participants are distributed in 4 waves of approximately 250 subjects, in which age, sex, and cognitive status are equally similarly distributed. As the distribution of those variables would be unknown at the time the randomization list is created, the resulting groups could have minimum size differences.

#### 2.10.3 Blinding

As with any other intervention studies on lifestyle habits, CITA Go On has limitations regarding the maintenance of masking of the group assigned to each participant. Nevertheless, participants are said in the randomization visit to be referred to Group 1 in the case of RHA and Group 2 in the case of MD-Int assignment. Therefore, participants are not actively told which group, intervention or control, they belong to. Blinded neuropsychologists perform the primary and secondary cognitive assessment, and participants are specifically requested to avoid discussing the intervention with the research staff and other participants. Primary outcome raters did not participate in any of the cognitive and socio-emotional intervention activities and have no access to randomization information in the database.

#### 2.10.4 Data collection and management

Data is collected in a computerized database using an electronic Clinical Record Form (eCRF) record system based on REDCap (Harris et al., 2008, 2018). REDCap is a secure web-based software platform designed to support data capture for research studies.

#### 2.10.5 Statistical methods for efficacy and secondary outcome analyses

First, the randomness of the samples will be verified to ensure that the baseline characteristics of the two groups do not show statistically significant differences. Univariate analysis will be conducted to identify sociodemographic and diagnostic variables that could differ between the two groups selected randomly before the study.

Continuous variables will be analyzed using Student’s t-test or the nonparametric Mann–Whitney test to compare medians when they do not follow a normal distribution. Categorical variables will be analyzed using the Chi-square or Fisher’s exact test for contrast in each case. Confounding variables will be included in the adjustment for all subsequent analyses. Logistic regression analysis will be performed to the control group and the MD-Int group. Composite scores will be calculated as *Z*-scores standardized to the Baseline mean and standard deviation (SD), with higher scores indicating better performance. The decline will be defined as a decrease in z-scores between Baseline and 24-month visit assessments (Z24month - Zbaseline < 0). Those covariables considered to influence changes in cognition will be introduced as model variables.

Third, mixed models will be used to study the trajectories of the different scores (NTBm) in the control and intervention groups. Mixed models explicitly account for the correlations between repeated measurements within each patient. The factors assumed to have the same effect across many patients are called fixed effects, and those likely to vary substantially from patient to patient are called random effects. In these mixed models, the interaction between the group variable (control vs. intervention) and the time will be included in all final analyses because the study’s final objective is to determine the effect of the intervention on effectiveness along time, adjusted for other variables that could influence them. For statistical analysis, the programs SPSS® (version 23) and R (version 4.2.3 or superior) will be used with a significance level of 95%.

#### 2.10.6 Cost-effectiveness analysis

A simulation model will be developed to represent the natural history of dementia to assess the cost-utility of the CITA GO-ON Study intervention (Mar et al., 2020). The model will compare two cloned cohorts that had slight differences in progression to dementia according to whether they received the multidomain intervention. It will calculate costs in Euros (€) in each cohort and utility in quality-adjusted life years (QALYs). Finally, the incremental cost-utility ratio (ICUR) will be estimated by dividing the incremental cost (Ca-Cb) by the incremental utility (Ua-Ub). The time horizon will be the lifetime of the patient. A 3% discount rate will be applied to costs and effectiveness. As the main economic impact of dementia is related to the care required as patients become dependent, a societal perspective will be used. Therefore, we will consider direct and indirect costs (Lopez-Bastida et al., 2006), Data for model parameterization will be mainly obtained from the CITA GO-ON study, although some parameters to build the model will be taken from the literature. The NTBm and functional scales (A-IADL-Q) will be used to obtain the model parameters that determine the progression of dementia from the pre-MCI stage to more advanced stages of the disease according to A-IADL-Q cut-off points (MCI < 60, mild dementia < 50 and moderate dementia < 40) (Dubbelman et al., 2022), die from the disease, or die from other causes. Mixed models for repeated measures allow the progression of the scales to be reproduced over time and will be used to simulate the trajectories of real patients (Mar et al., 2020). The model will reflect the improvement introduced by the effectiveness of the intervention in patients to the extent that those treated with multimodal intervention delay their evolution to dementia and improve their health-related quality of life score. We have constructed a preliminary version of the model using a synthetic database. The result has been cross-validated with other nine models within the International Pharmaco-Economic Collaboration on Alzheimer’s Disease (IPECAD) collaboration (Handels et al., 2025).

## 3 Results

In July 2021, the CITA GO-ON trial was launched, along with an educational campaign targeting healthy aging practices in the Gipuzkoa region. The campaign was directed towards the capital city of San Sebastian and three smaller municipalities, Deba, Beasain, and Irun. An informational initiative was reached through the direct mail of over 110,000 citizens aged 60 years and above. A robust media presence, including local newspaper advertisements and radio segments, complemented the campaign. Moreover, up to 30 presentations were delivered to neighborhood associations in the specified locales, with a focus on healthy aging and dementia prevention.

**Figure 3:**
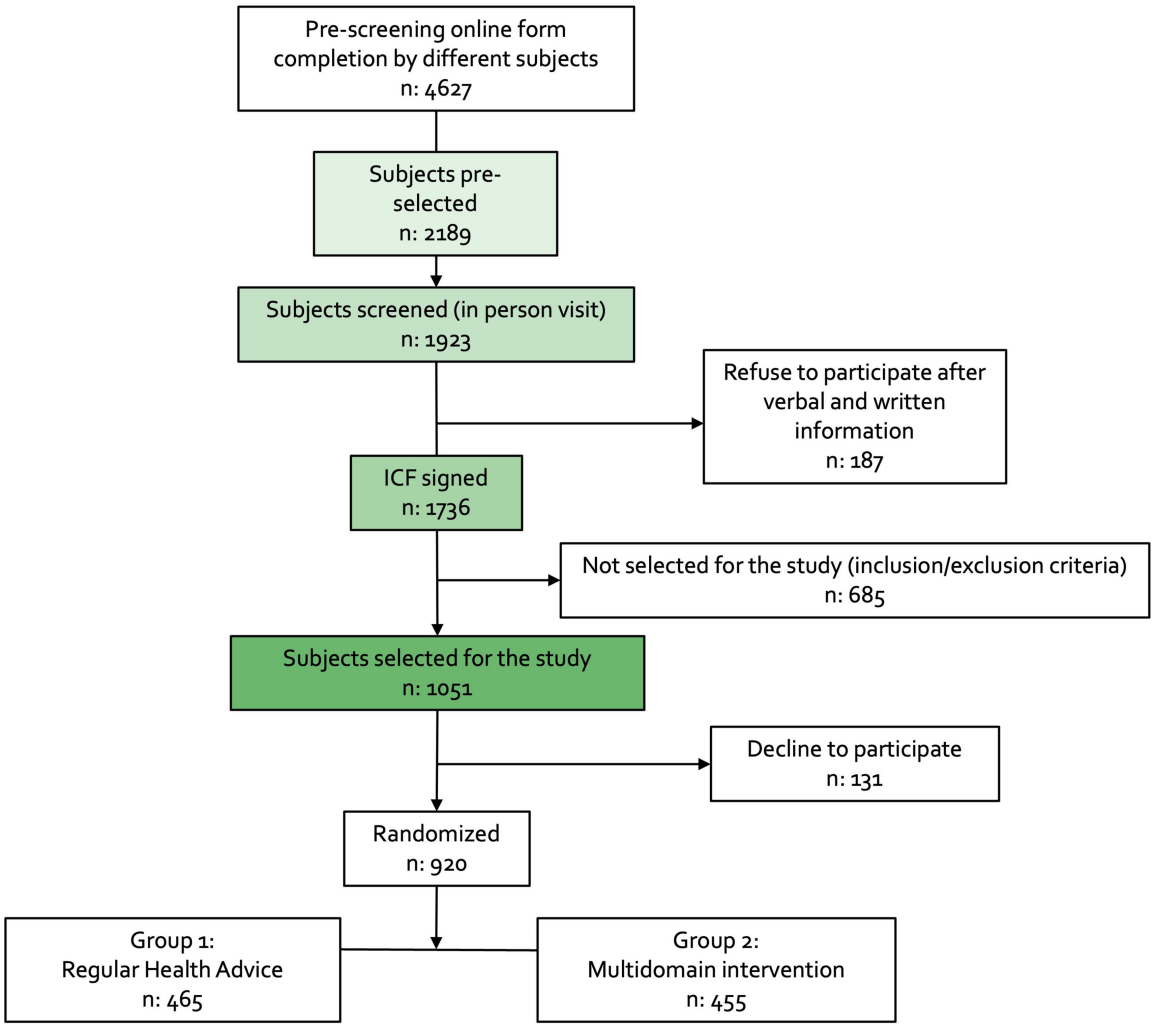
CITA GO-ON study recruitment process CONSORT flowchart.

The campaign culminated in January 2024, with a preliminary screening of 4,627 individuals via an online survey. Of these, 2,189 people were selected for an in-person screening visit. By this moment, 1736 participants were informed about the study and signed an informed consent form. Of these, 1,051 (60%) were selected for the study (Figure 3). The recruitment phase has concluded in autumn 2024. There were no significant differences (*p* = 0.062) between participants’ self-reported mean CAIDE dementia risk score calculated using the online form [mean, (SD); 7.23 (1.77)] and the in-score calculated at the screening visit [mean, (SD); 76.98 (1.70)]. This finding indicates that the online form may be a reliable tool for assessing the risk of dementia.

In the ongoing CITA GO-ON trial Intervention period, 465 individuals have been allocated to the Group 1-Regular Health Advice (RHA) group, and 455 have been assigned to the Group 2-Multidomain Intervention (MD-Int) group. Baseline assessments revealed that a substantial majority (approximately 95%) of the participants randomly selected for the trial exhibited modifiable dementia risk factors as identified by their Cardiovascular Risk Factors, Aging, and Dementia (CAIDE) score.

The characteristics of the already screened participants are shown in Table 5. The mean (SD) age of the 68.67 (6.17) of subjects who were screened was 880 (50.7%) women. Selected participants were older and had a higher risk of dementia, as measured by the CAIDE dementia risk score, compared to not selected subjects. Specifically, they had higher systolic blood pressure and body mass index, were less physically active, performed poorly on brief cognitive screening tests, and reported more frequent memory complaints.

**Table 5:** Age, sex, CAIDE dementia risk score, and brief cognitive tests score of the interested subjects depending on their selection status.

	**Total (*n*: 1736)**	**No selected (*n*: 685)**	**Selected (*n*: 1051)**	*p-value*
Age (years), x(sd)	68.67 (6.17)	67.16 (5.53)	69.65 (6.36)	<0.001
Sex: woman, *n* (%)	880 (50.7)	365 (41.5)	515 (58.5)	0.076
CAIDE dementia risk score	7.16 (1.67)	6.48 (1.786)	7.62 (1.427)	<0.001
Education (years)	13.65 (4.60)	14.87 (4.34)	12.86 (4.60)	<0.001
Systolic blood pressure (mmHg),x (sd)	141.45 (18.55)	137.59 (18.89)	143.99 (18.40)	<0.001
Serum total cholesterol (mmol/l), x (sd)	5.53 (1.09)	5.53 (1.06)	5.53 (1.10)	0.919
Body mass index (kg/m2), x (sd)	23.76 (8.83)	23.03 (8.67)	24.24 (8.92)	0.008
Body mass index > 30 kg/m2, *n* (%)	371 (21.4)	109 (29.4)	262 (70.6)	<0.001
Physicaly inactive, n (%)	633 (36.4)	204 (32.2)	429 (67.8)	<0.001
Fototest, x (sd)	39.55 (5.80)	41.99 (5.03)	38 (5.73)	<0.001
Memory Alteration Test, x (sd)	41.98 (5.34)	44.55 (3.69)	40.35 (5.58)	<0.001
Cognitive Change Index (memory items), x (sd)	21.05 (7.93)	16.95 (5.90)	23.67 (7.96)	<0.001

## 4 Discussion

The CITA GO-ON Study is a lifestyle intervention efficacy trial conducted in Southern Europe, which aims to replicate the findings of the FINGER study and ascertain its reproducibility across diverse global populations. It is part of the broader World-Wide FINGERS network (Kivipelto et al., 2018b, 2020), which includes similar initiatives in Latin America (Crivelli et al., 2023), Singapore (Chew et al., 2021), the United States (Baker et al., 2024) and others (Moon et al., 2025; Röhr et al., 2021; Sugimoto et al., 2021; Deckers et al., 2024; Feldman et al., 2023; Gardener et al., 2024; Kumagai et al., 2023). The CITA GO-ON study’s main protocol emanates from a collaborative effort among leading researchers and institutions within our region in each of the intervention domains and the exploratory variables of the study.

In alignment with the FINGER trial methodology (Ngandu et al., 2014), the CITA GO-ON study mirrors its inclusion protocols and outcomes yet diverges in its recruitment strategy. Unlike the FINGER trial, which relied on prior health registries, recruitment in the CITA GO-ON has been community-based. This population-oriented approach is an efficient strategy for identifying adults with modifiable risk factors of dementia and cognitive decline. Our delineation of a “cognitively frail” population was based on the combination of an increased dementia risk profile, as measured by the CAIDE risk score and the presence of early cognitive changes including MCI. These are characterized by relevant subjective memory complaints corroborated by consistent accounts or cognitive performance that falls short of expectations in brief cognitive assessments. Based on this approach, we already observed in the GOIZ ZAINDU pilot study that the greatest effect on cognition is produced in executive and processing speed function, which is consistent with previously reported data from the FINGER trial. We understand that a more prolonged intervention period and a larger sample study will might help us to expand this evidence.

Previous lifestyle intervention studies aimed at dementia prevention underscored the critical nature of participant selection (van Charante et al., 2016; Andrieu et al., 2017), indicating that unselected participants may harbor a similar dementia risk profile to their selected counterparts (Licher et al., 2023). Consequently, by adhering to stringent inclusion criteria, the CITA GO-ON study ensured that the chosen participants exhibited elevated scores on the CAIDE dementia risk index and inferior results on brief cognitive tests (Table 5).

The main protocol of the CITA GO-ON trial, which does not exhaustively delineate them, is comprehensive enough to accommodate ancillary studies. These potential studies will investigate various topics, including alterations in fluid biomarkers, the effects of APOE genotype, blood DNA methylation, sleep quality and quantity and, and a preliminary assessment of the influence of virtual coaches on follow-up.

Blood sample collection and neuroimaging acquisition are conducted in a subgroup of participants who have consented to it (Figure 2). At this point, we have obtained over 700 MRI and blood samples from study participants. This representative subgroup of the study will help us analyze multimodal biomarkers (biofluids and structural neuroimaging). The aim is to evaluate the intervention’s biological impact and identify subjects with a specific clinical-biological phenotype who will benefit most from the intervention. The biofluid samples are not limited to plasma or epigenetic markers. They will include proteomic analyses and explore future options such as metabolomics, genomics, or biomarkers of vascular/synaptic damage. MRI acquisition pre-and post-intervention will assess brain changes, with the possibility of integrating other methods in later phases. This effort is made to create a multimodality unified platform that combines clinical data, biomarkers, and “omics” strategies to analyze the interaction between modifiable risk factors and pathophysiological processes in people at risk of dementia.

### 4.1 Limitations

There are some inherent limitations to this type of trials that we have tried to overcome with the study design. Recruitment from a single center for the required sample size represents a significant challenge, demanding innovative strategies to enhance communication and dissemination activities, which are not typically within the realm of clinicians and researchers. This manuscript underscores such innovative strategies, as engaging directly with the target population has led to the development of a more effective study protocol tailored to the specific needs and demands of the population. To ensure the project’s sustainability and the efficient use of resources, the participants were allocated into four waves of 250 individuals for the randomization phase, thereby optimizing resource allocation and preventing resource dilution during periods of low recruitment and overburden during high participants turnout.

Another limitation regarding the recruitment process is the healthy volunteer bias. For that reason, we have included the presence of modifiable dementia risk factors as inclusion criteria; for that reason, we have lost approximately 45% of the interested subjects (Figure 3). Finally, the online prescreening questionnaire may have lost older adults who are digitally illiterate. However, we have made great efforts to avoid it, providing a telephone number in the mass mailing as an alternative. In order to reach communities with poorer communications or poorer accessibility, we also scheduled community talks in rural areas. One of the most challenging issues to achieve in this type of study is blinding. To reduce the impact of this factor, the neuropsychologists responsible for evaluating the primary outcome were not aware of participant assignment to intervention groups, and participants were encouraged not to comment on program details during study visits.

Considering the duration of the project, loss to follow-up was anticipated. The intervention activities were crafted with the participant perspectives in mind, encompassing user-friendly and engaging initiatives, such as the VIVIFRAIL© multicomponent tailored physical activity program. Cognitive training materials called EXERCITA® have also been developed to consider the Basque Country’s cultural and linguistic nuances. Nutritional and culinary workshops led by renowned local chefs and nutritionists aim to provide healthy and appealing culinary skills. In addition to fostering the promotion of socio-emotional skills, the socio-emotional intervention also plays a transversal role in enhancing social commitment, motivation, and adherence to the program. Each follow-up visit dedicated a segment to reiterating the study primary objectives to the participants, addressing any uncertainties or challenges they may have encountered or anticipated.

## 5 Conclusion

The overarching aim of the CITA GO-ON trial, bolstered by the World-Wide FINGERS consortium, is to elucidate the strategies needed to achieve an active, healthy, and dementia-free aging process. The design of the intervention is predicated on the principle of scalability and ease of implementation, so that upon demonstration of efficacy, it could be readily integrated into public healthcare systems.

## Data Availability

The raw data supporting the conclusions of this article will be made available by the authors, without undue reservation.

## Footnotes

^1^ https://www.cita-alzheimer.org/es/cita-go-on#formu-citago

^2^ http://vivifrail.com/resources/

## Glossary

FINGER: Finnish geriatric intervention study to prevent cognitive impairment and disability
GZ: GOIZ ZAINDU
CAIDE: cardiovascular risk factors, aging and dementia
MD-Int: multidomain intervention
RHA: regular health advice
MCI: mild cognitive impairment
NTB: neuropsychological test battery
NTBm: modified neuropsychological test battery
WHO: World Health Organization
MAPT: multidomain Alzheimer preventive trial
PreDIVA: prevention of dementia by intensive vascular care
T@M: memory alteration test
CCI: cognitive change index
SCD: subjective cognitive decline
DSM-IV: diagnostic and statistical manual of mental disorders, 4th edition
GGP: general practitioners
SD: standard deviation
GADS: Goldberg anxiety and depression scale
REDCap: research electronic data capture
HRQL: health-related quality of life
SiMoA: single molecule array
SPPB: short physical performance battery
A-IADL-Q: The Amsterdam questionnaire for instrumental activities of daily living
EQ-D5-5L: EuroQol 5 dimensions
TMMS: trait meta-mood scale
UCLA: University of California at Los Angeles - loneliness
SWLS: satisfaction with life scale
MAAS: mindful attention awareness scale
CDR-risk: Connor-Davidson Resilience scale risk
PSQ: perceived stress questionnaire
PREDIMED: PREvención con DIeta MEDiterránea (prevention through Mediterranean diet)
CONSORT: consolidated standards of reporting trials
