# Supplementary material for: CITA GO-ON study. A community based multidomain lifestyle intervention to prevent cognitive decline. Protocol design and recruitment process
Source: Front Aging Neurosci. 2025 Jun 16;17:1539711. doi: 10.3389/fnagi.2025.1539711 (PMC12206835; doi:10.3389/fnagi.2025.1539711)
Supplement: Supplementary file 1 [file Data_Sheet_1.docx]

**SUPLEMENTARY MATERIAL:**

Table S1: CITA GO-ON exclusion criteria.

| **Exclusion criteria** |
| --- |
| 1. Inability to perform a neuropsychological evaluation or a cognitive stimulation program (sensory limitation, mental retardation, illiteracy) |
| 1. Previous diagnosis of any neurological, psychiatric or systemic disease that produce cognitive impairment or dementia including, but not limited to, Huntington's disease, multiple sclerosis, Parkinson's disease, Down syndrome, alcohol abuse or active drugs, or major psychiatric disorders including ongoing major depression, schizophrenia or bipolar or schizoaffective disorder. |
| 1. Unstable ischemic cardiopathy, uncontrolled heart arrhythmia, thromboembolic disease in the last year. Moderate cardiorespiratory insufficiency (including Class III or IV congestive heart failure, clinically significant aortic stenosis, cardiac arrest history, or uncontrolled angina). Currently receiving physical therapy or cardiopulmonary rehabilitation. |
| 1. Any cerebrovascular accident with significant residual effects on cognition or functional autonomy. |
| 1. History within the last two years of treatment for primary or recurrent malignant disease, excluding non-melanoma skin cancers, resected cutaneous squamous cell carcinoma in situ, basal cell carcinoma, cervical carcinoma in situ, or situ prostate cancer with normal prostate-specific antigen posttreatment |
| 1. History of hip fracture, joint replacement, or spinal surgery in the last six months |
| 1. Barthel index score < 90. |
| 1. Geriatric Depression Scale ≥ 9 points. |
| 1. Large vessel stroke in the past two years |
| 1. History of transient ischemia attack (TIA) or minor stroke in the last six months |
| 1. Recent (< 3 months) bone fracture. |
| 1. Dementia or Moderate Cognitive impairment at baseline evaluation |
| 1. Clinically significant abnormalities in laboratory blood tests as per judgment of the site Study Clinician |
| 1. Any conditions affecting safe engagement in the intervention in the judge of the study investigators. |

Table S2. English language version of the prescreening online questionnaire content.

| **WHAT IS THE "CITA GO-ON INITIATIVE"?**  CITA-Alzheimer launches an initiative to promote active and healthy aging and prevent cognitive decline and dementia.  The initiative, where the critical element to carry it out are people who, like you, show interest in this project, consists of two parts: 1) a research study on lifestyle habits to prevent dementia and 2) a questionnaire to explore how the COVID-19 pandemic has affected the lifestyle and health of middle-aged and older people.  Epidemiological and demographic studies have raised the alarm about the consequences of the unstoppable phenomenon of population aging. Suppose innovative, affordable, and proven measures are not taken. In that case, the number of people in a situation of dependency due to diseases and socio-economic conditions associated with aging will be uncountable in the coming decades.  Getting older and getting older is an apparent risk factor for pathologies and personal and social conditions that induce loss of autonomy and dependence. The decrease in social interaction, cognitive deterioration, and dementia are a clear exponent of this well-known biological-pathological phenomenon. Thus, sufficient knowledge and scientific evidence have been accumulated to affirm that the biological and social trajectory of aging has a plastic character and that a good part of the deviations that occur towards diseases are susceptible to prevention and modification through lifestyle interventions. One in three cases of dementia in the world is preventable. The risk of developing dementia can be up to 60% lower in people who follow what are known as "healthy lifestyle habits."  For all these reasons, it is essential to know how to take care of our brain health to improve the quality of life as individuals and reduce the prevalence of dementia as a society.  **WHAT DOES THE "CITA GO-ON RESEARCH STUDY" CONSIST OF?**  The CITA GO-ON study is a research project that seeks to promote healthy lifestyle habits and control vascular risk factors. It also tries to demonstrate if these practices improve cognitive ability and prevent deterioration in people at increased risk of dementia.  To study the effect of this lifestyle intervention, the participants will be divided into two groups: one group will follow the recommendations, care, and care measures approved and already available, and the other will participate in a more intense program with individual visits and group workshops. The intervention period lasts two years. During this period, control of risk factors, personalized physical exercise programs, cognitive training, adoption of healthy dietary habits, and promotion of emotional intelligence and the awareness of the impact of emotions in cognition will be worked on.  All participants must attend evaluations before, at the middle, and at the end of the intervention period.  For more information regarding the CITA GO-ON initiative, please visit our web page: <https://www.cita-alzheimer.org/> |
| --- |
| **ONLINE FORMULARY**  Please complete the following questions. This part will not take you more than 2 minutes to complete. If you have them on hand, we advise you to have your cholesterol and blood pressure data within reach. If you do not have this data or are older than six months, you can measure them at any pharmacy.  All questions in this first part must be completed to continue with the questionnaire.   1. **Sociodemographic data:**  - Age: - Sex:   **2. Anthropometric data, years of education, physical exercise, and cardiovascular risk factors to estimate CAIDE dementia Risk Score:**   - How many years did you study after the age of six? 🞏 0-6 🞏 7-9 🞏 More than 10 - High (m):___________ - Weigh (kg): ___________ - Have you ever been told you have hypertension? Do you take medication for it, or do you usually have high blood pressure? 🞏 Yes 🞏 No - Have you ever been told you have high cholesterol? Is your total cholesterol greater than 250 mg/dl? 🞏 Yes 🞏 No - Do you usually do physical exercise for 20-30 minutes a week that makes you sweat or speeds up your breathing? 🞏 SI 🞏 No   **3. Medical history:**  Please check only the boxes that apply to you:   - Do you have vision or hearing problems preventing you from doing activities such as reading or following a conversation without difficulty? 🞏 Yes 🞏 No - Do you need help to carry out your usual activities (eating, personal hygiene, dressing, housework, going outside)? 🞏 Yes 🞏 No - Do you have difficulty walking without assistance for 20 minutes at a time? 🞏 Yes 🞏 No - Have you suffered a heart attack or other thrombotic disease in the last year? 🞏 Yes 🞏 No - Have you suffered a stroke or cerebrovascular disease in the last two years that caused physical or mental consequences? 🞏 Yes 🞏 No - Have you been diagnosed with any of the following diseases?   - Dementia or moderate cognitive impairment 🞏 Yes 🞏 No   - Parkinsonism or Parkinson's Disease 🞏 Yes 🞏 No   - Bipolar disorder 🞏 Yes 🞏 No   - Heart failure 🞏 SI 🞏 No   - Cancer or neoplastic disease in the last two years 🞏 Yes 🞏 No   **4. Other data:**  Are you used to making video calls? 🞏 Yes 🞏 No  If you marked "yes":  Which of the following apps do you know or have you used?   - Zoom - Whatsapp - Skype - Other (specify which):   What device do you use for video calls?   - Phonel - Tablet - Computer   If you marked no", specify the reason:   - I do not like this kind of communication. - I don't know how to do it. - I don't have any device to do it. |
| **Thank you very much for being part of the CITA GO-ON initiative!**  We will email you in the coming weeks if you have been selected to participate in the CITA GO-ON research study. |

Table S3: CITA GO-ON study cognitive variables and composites.

|  | **COGNITIVE COMPOSITES** ^a^  *(Minimum number of tests required to index calculation/total)* | | | | |
| --- | --- | --- | --- | --- | --- |
|  | Global | EF | PS | MRY | VPC |
| **TESTS** | (8/14) | *(3/5)* | *(2/3)* | *(3/6)* | *(3/3)* |
| CERAD Category Fluency | X | X |  |  |  |
| WMS-R Digit Span (total) | X | X |  |  |  |
| CST (Condition C) | X | X |  |  |  |
| TMT shifting score (B-A) | X | X |  |  |  |
| Stroop test interference score (3-2) | X | X |  |  |  |
| Letter and Number (WAIS-III)^b^ | X* | X* |  |  |  |
| Letter Digit Substitution Test | X |  | X |  |  |
| CST (condition A) | X |  | X |  |  |
| Stroop test (condition 2) | x |  | X |  |  |
| WMS-III Logical Memory (immediate) | X |  |  | X |  |
| WMS-III Logical Memory (delayed) | X |  |  | X |  |
| CERAD Word List Learning | X |  |  | X |  |
| CERAD Word List Recall | X |  |  | X |  |
| WMS-R Visual Paired Associates (immediate) | X |  |  | X |  |
| WMS-R Visual Paired Associates (delayed) | X |  |  | X |  |
| Judgement Line Orientation |  |  |  |  | X |
| Rey Complex Figure Copy |  |  |  |  | X |
| 15 Object Test |  |  |  |  | X |

Abbreviations: EF= Executive Function, PS= Processing Speed index, MRY= memory, VPC= Visual Perception and Construction, CERAD = Consortium to Establish a Registry for Alzheimer's Disease, WMS-R = Wechsler Memory Scale-Revised, CST = Concept Shifting Test, TMT = Trail Making Test, WAIS-III = Wechsler Adult Intelligence Scale-III.

^a^ Five cognitive domain composites will be created: Z scores will be standardized to the baseline mean and standard deviation.

^b^ Letter and Number (WAIS-III) is being applied to have a TMTB comparison measure in case a telematic administration was needed.

Table S4. Brain MRI sequences (time of adquisition) obtained at baseline and 24-month visit to evaluate brain volume trajectories, cortical thickness, and white matter changes (MRI).

| - AAHScout_32ch_ISO (0’28’’) |
| --- |
| - low resolution single shot whole brain (4’5’’) |
| - MPAGE_1X1X1_iso (9’50’’) |
| - FLAIR - t2_tirm_tra_dark-fluid_3mm (2’44’’) |
| - SWI - t2_fl3d_tra_p2_swi (5’43’’) |
| - MPRAGE_1.25_iso (7’23’’) |
| - ASL 1 medida - ep2d_tra_pasl_ (0’20’’) |
| - ASL 63 medidas- ep2d_tra (5’30’’) |
| - gre_field_mapping_DTI_2x2x2 (2’28’’) |
| - DWI (diffusion)- ep2d_diff_mddw_2x2x2_122 (8’59’’) |
| - gre_field_mapping_EPI_RS_2x2x2 (1’26’’) |
| - resting state functional sequence ep2d_rest_state_TE25_38_CORTES_210 (10’54’’) |

Figure S1. Environments to promote cognitively active habits.

*In our daily lives, we can use numerous tools to stay cognitively active. In the guidelines, we will give you tips on how to take advantage of these daily life tools to stay cognitively active. We will present you habits and recommendations for each area of daily life, known as “environments”, and provide you with the required materials to carry out these recommendations. This represents an approach to initiate cognitive stimulation.*


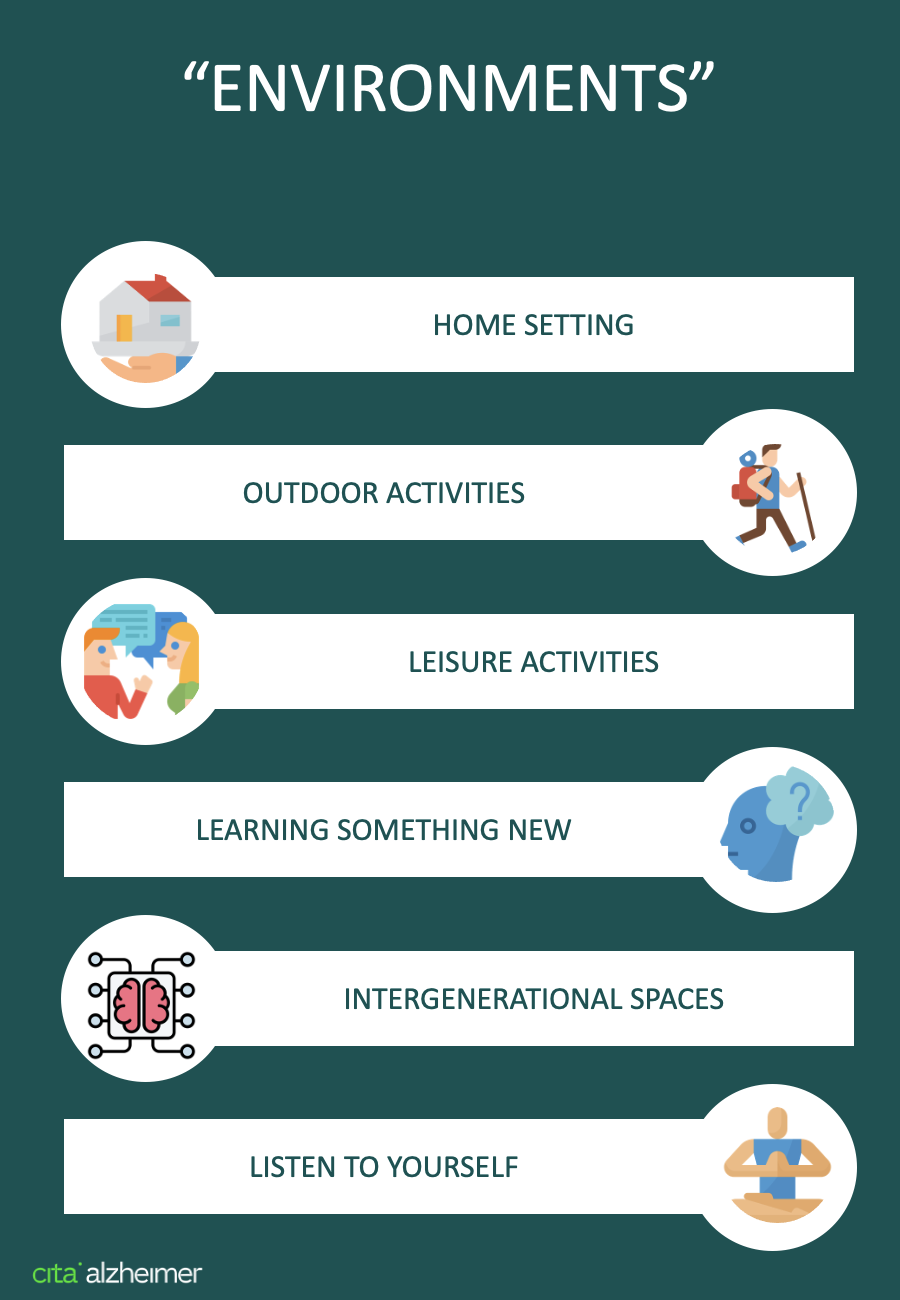


Figure S2. Home setting.

*Daily routines at home and home duties offer opportunities to enhance planning skills and working memory. In this section, several examples are provided.*

Figure S3. Intergenerational spaces.

*Generational intelligence is a concept that can be defined as the ability to understand how people from generations other than ours perceive the world. This allows us to reinforce the aspects of behavior and decision making through this understanding. Initiatives in different environments and contexts that promote the creation of “intergenerational spaces” to share experiences bring benefits to young generations, improving the acquisition of knowledge and social skills, and also to older participants, reducing feelings of loneliness and improving emotional aspects. On the other hand, it prevents fragile people from risky situations such as isolation and detriment to their quality of life.*
